# Improvement in cerebral oxygen saturation with sinus conversion during off pump coronary artery bypass graft: A case report

**DOI:** 10.1097/MD.0000000000033495

**Published:** 2023-05-17

**Authors:** He Won Hwang, Jin Ho Kim, So Woon Ahn

**Affiliations:** a Department of Anesthesiology and Pain Medicine, CHA Bundang Medical Center, Seongnam-si, Gyeonggi-do, South Korea; b Department of Anesthesiology and Pain Medicine, CHA Bundang Medical Center, CHA University, Seongnam-si, South Korea.

**Keywords:** atrial fibrillation, near-infrared spectroscopy (NIRS), sinus conversion

## Abstract

Near-infrared spectroscopy (NIRS) is a noninvasive bedside tool for monitoring regional cerebral oxygen saturation (rSO_2_). The sinus conversion of atrial fibrillation (AF) was shown to be responsible for increasing rSO_2_. However, the reason for this improvement has not yet been clearly explained.

**Patient concerns:**

We report the case of a 73-year-old woman who underwent cardioversion during an off-pump coronary artery bypass under NIRS and live hemodynamic monitoring.

**Interventions:**

Unlike previous studies that failed to control and compare all conditions during procedures, this case showed real-time fluctuating hemodynamic and hematological values, such as hemoglobin (Hgb), central venous pressure (CVP), mean arterial pressure (MAP), cardiac index (CI), left ventricular end-diastolic pressure (LVEDP), and SVO_2_.

**Outcomes:**

The rSO_2_ increased immediately after cardioversion and decreased during the obtuse marginal (OM) graft and after AF was obtained. However, no other hemodynamic data showed the same or opposite directional changes in the rSO_2_.

**Lessons:**

Significant instantaneous changes were observed in rSO_2_ using NIRS after sinus conversion, without obvious hemodynamic alterations in the systemic circulation or other monitoring values.

## 1. Introduction

Maintenance of brain perfusion is crucial and a major outcome factor in various acute disorders in which atrial fibrillation (AF) is likely to occur, such as stroke and sepsis. AF has been shown to worsen the prognosis of stroke, sepsis, and myocardial infarction.^[1–4]^ Furthermore, and recent trials have found that AF is associated with cognitive impairment independent of cerebral infarction^[1,4,5]^ and is an independent predictor of cognitive dysfunction ranging from cognitive impairment to dementia.^[6]^ Brain hypoperfusion during AF has been identified as a major contributing factor to cognitive decline.^[4,5,7]^ However, no precise pathophysiological mechanism has been identified, nor the extent to which AF itself affects perfusion.

Near-infrared spectroscopy (NIRS) is a noninvasive bedside tool for monitoring regional cerebral oxygen saturation (rSO_2_). We report the case of a 73-year-old woman who experienced an instantaneous increase in rSO_2_ after sinus conversion during an off-pump coronary artery bypass under live hemodynamic monitoring.

## 2. Case report

**Figure 1.**
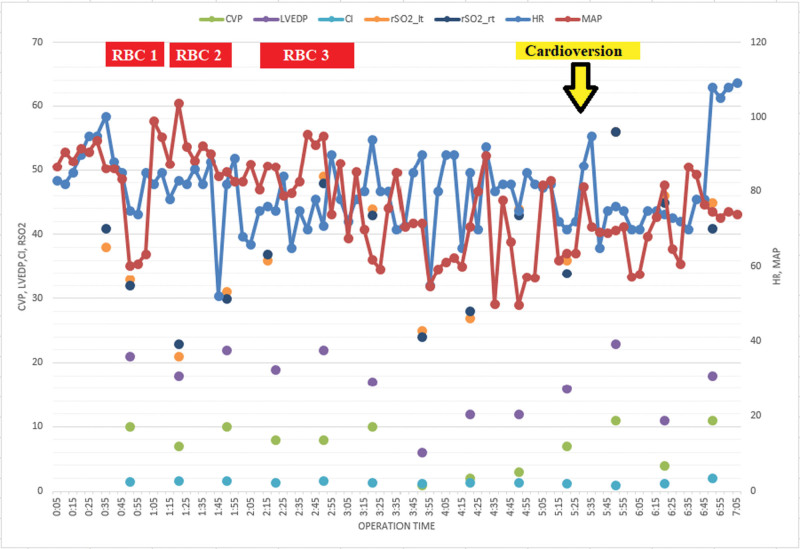
Hemodynamic and near-infrared spectroscopy (NIRS) score during anesthesia.

A 73-year-old woman with a history of hypertension and coronary artery occlusive disease with balloon angioplasty 17 years prior, presented to the emergency department with shortness of breath. Emergency coronary angiography revealed a left main coronary artery occlusion of up to 90% with thrombi-like haziness, 95% LN LCX, and 70% LN pRCA with luminal haziness. Emergency off-pump coronary bypass graft surgery was performed for acute myocardial ischemia. The initial hemoglobin (Hgb) was 8.7 g/dL, central venous pressure (CVP) was 10 mm Hg, and rSO_2_ were 21 and 23 in the left and right halves, respectively (Table 1). The electrocardiography result was AF and ST depression. During surgery, we continuously monitored the cardiac index (CI), left ventricular end-diastolic pressure (LVEDP), mixed venous oxygen saturation (SVO_2_), CVP, end-tidal CO_2_ (EtCO_2_), and rSO_2_ (INVOS; Medtronic, Minneapolis, MN) and analyzed blood gas hourly. The INOS system uses 4 wavelengths that are absorbed by Hgb (730 and 810 nm) and an algorithm with 25:75 arterial/venous values. After induction of anesthesia, transesophageal echocardiographic examination confirmed the absence of an intracardiac thrombus. Even though PaO_2_, PaCO_2_, and mean arterial pressure (MAP) were within the normal limits, the initial rSO_2_ was far below the normal range (left/right: 21/23, Table 1); therefore, we decided to transfuse packed red blood cell (pRBC).^[8,9]^ One unit of pRBC transfusion increased the rSO_2_ values up to 48% and 30%, respectively, and rSO_2_ increased up to 110% and 86% from baseline and Hgb to 10.2 g/dL after 3 units of pRBC transfusion (Table 1). However, MAP, CVP, and LVEDP values fluctuated during diagonal, left anterior descending vessel grafting (Fig. 1).

**Table 1:** Hemodynamic and hematologic data on each surgical time point.

	Post induction	LIMA harvest	LAD graft	RCA graft	Sinus conversion	OM graft	Return to A fib
rSO_2_ Lt	21	31	44	36	56	46	45
rSO_2_ Rt	23	30	43	34	56	45	41
ΔrSO_2_ L/R		48/30	42/43	−18/−21	56/65	−18/−20	−2/−9
MAP	60	104	62	64	70	82	75
HR	75	83	80	87	75	70	108
CVP	10	7	10	7	11	11	11
LVEDP	21	18	22	19	23	23	18
CI	1.5	1.6	1.4	1.2	1	1.3	2
EtCO_2_	31	32	32	27	31	28	33
PaO_2_	242	138	174		180		
Hgb	8.7	7.7	10.2		10.9		
Transfused pRBC(total)		2	1 (3)	(3)	(3)	(3)	(3)

CVP = central venous pressure, Et CO_2_ = end tidal CO_2_, % change from prior time point, Hgb = hemoglobin, LVEDP = left ventricular end diastolic pressure, MAP = mean arterial pressure, pRBC = packed red blood cell, rSO_2_ = regional cerebral oxygen saturation.

**Figure 2.**
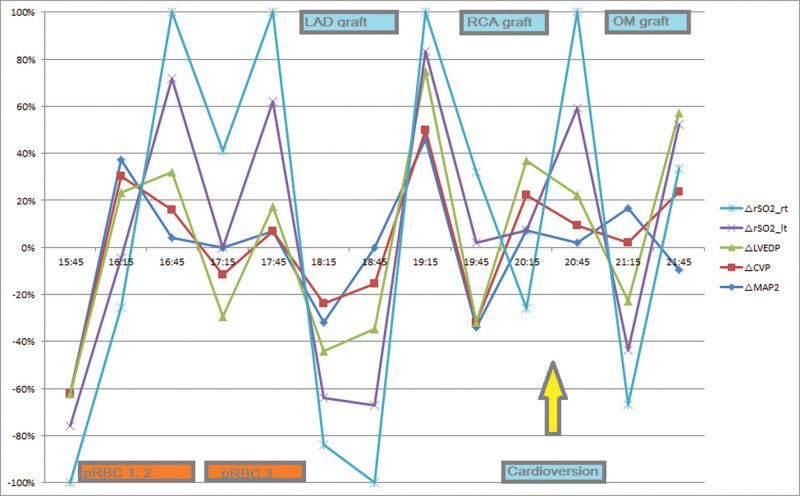
Hemodynamic and NIRS changes during operation. NIRS = near-infrared spectroscopy.

During aorta-to-right coronary artery grafting, cardioversion was performed to obtain sinus rhythm. After sinus conversion, CVP was higher and MAP was lower than before cardioversion; however, rSO_2_ instantaneously increased to56 (56%) left and 56 (65%) right, which were the highest values obtained during the intraoperative period (Fig. 1 and Table 2). Other parameters that might influence cerebral perfusion such as Hgb, PaCO_2_, and PaO_2_ remained unchanged. During obtuse marginal (OM) grafting, MAP fluctuated from 57 to 82 mm Hg; however, rSO_2_ was stable. After sternal closure, the rhythm returned to AF, and rSO_2_ returned to the value before cardioversion (Fig. 1). The rSO_2_ increased immediately after cardioversion and decreased during the OM graft and after AF was obtained. However, no other hemodynamic data showed the same or opposite directional changes in the rSO_2_ (Table 2 and Fig. 2).

**Table 2:** Hemodynamic and NIRS changes after sinus conversion.

	After sinus conversion	OM graft	After returning to A fib
rSO_2_ Lt	36→56 (36)	→46 (−18)	→45 (−2)
rSO_2_ Rt	34 → 56 (65)	→45 (−20)	→41 (−8)
MAP	64→82 (28)	→70 (−15)	→75 (7)
HR	72→74 (2)	→76 (3)	→108 (54)
CVP	7→4 (−75)	→11 (175)	→11 (0)
LVEDP	16→11 (−45)	→23 (190)	→18 (−22)
CI	1.2→ 1.3 (8)	→1 (23)	→2 (100)

() = % change from prior time point, CI = cardiac index, CVP = central venous pressure, HR = heart rate, LVEDP = left ventricular end diastolic pressure, MAP = mean arterial pressure, NIRS = near-infrared spectroscopy, rSO2 = regional cerebral oxygen saturation.

## 3. Discussion

NIRS measures light absorbance to calculate cerebral oxygen saturation of the frontal cortex (rSO_2_), representing mixed cerebral arterial and venous oxygen saturation.^[10]^ It also provides the status of cerebral hemodynamics in multiple pathological processes.^[11,12]^ The level of rSO_2_ depends on the balance between cerebral oxygen supply^[13]^ and demand.^[14]^ Several factors that influence cerebral oxygen saturation have been described.^[15–17]^

Several investigations have shown that electrical cardioversion or catheter ablation increases cerebral oxygen saturation.^[18,19]^ However, the reason for the improvement has not been clearly explained. These studies did not observe patient data continuously and only compared basic parameters such as SaO_2_, MAP, and HR, with NIRS at pre and post-procedural time points. In this case, unlike in other studies, we continuously monitored MAP, CVP, PAP, CI, SVO_2,_ or other critical invasive hemodynamic parameters simultaneously. The significant increase in bilateral rSO_2_ with the restoration of sinus rhythm is in accordance with previous studies that showed an improvement in microvascular function after cardioversion, which was independent of blood pressure changes.^[20,21]^ Notably, the change occurred immediately after sinus conversion, even though there were no changes in the relevant direction of Hgb, CVP, MAP, CI, LVEDP, SVO_2_ and other monitored parameters that could be related to cerebral blood flow or perfusion pressure. Furthermore, compared with LAD grafting, RCA and LCA grafting during off-pump coronary artery bypass caused more distortion of the heart; Therefore, in most cases, there were profound changes in vital signs, especially during OM grafting. However, cardioversion was performed between the RCA and OM grafts and the rSO_2_ immediately increased to 56 and 65%, respectively (Table 1). The increased rSO_2_ was maintained throughout OM grafting at the same level as that during the LAD grafting period.

Significant changes in rSO_2_ were observed with pRBC transfusion and sinus conversion. An rSO_2_ level <50% is considered pathological and is associated with worse outcomes.^[22]^ Most authors state that a 75% to 80% decrease from baseline under intervention (e.g., anesthesia) or an absolute decrease below 50% should be avoided.^[22,23]^ Red cell transfusions are intended to improve oxygen delivery to tissues. In this patient, the initial left rSO_2_ was 21 and the right was 23; although MAP and PaO_2_ were within the normal ranges, we decided to transfuse 2 pRBC. This resulted in an increase of 48%(left)/30%(right), and additional pRBC transfusion led to further improvement in the NIRS score (42/43%; Table 1 and Fig. 2). However, Hgb (10.2 g/dL) was above the target level for patients with acute coronary syndrome,^[24]^ and the NIRS value did not improve by >50%. The changes in NIRS values in our case, in terms of AF and sinus rhythm, were as expected after pRBC transfusion (changes in rSO_2_ 48/30% and 56/65% from the prior time point for the right and left-side, respectively; Table 1).

Although RBC transfusion increases oxygen-carrying capacity, which improves cerebral oxygenation, we could not explain how sinus conversion improved cerebral oxygenation, even with numerous continuous live monitoring procedures. Unlike previous studies that failed to control and compare all conditions such as Hgb and blood pressure during procedures, this case observed real-time fluctuating hemodynamic and hematological values and confirmed that the increase was maintained regardless of what was expected to affect rSO_2_. AF is known to reduce total cardiac output by approximately 20%. However, in this case, it was not observed immediately after the sinus conversion. A recent study evaluated the impact of AF and sinus rhythm on cerebral blood perfusion using computational simulation and reported that the mean cerebral flow rates in AF and sinus rhythm were similar, even when considering cerebral autoregulation. The authors concluded that flow variability was higher in AF than in sinus rhythm, with a peak at arteriolar and capillary levels, resulting in local hypoperfusion.^[25]^ It may be the mechanism behind the improvement in the patient NIRS. Therefore, factors other than the monitoring numbers listed in this case are thought to be involved in the direct and instantaneous cerebral perfusion improvement, and further research on this may be necessary.

Furthermore, because MAP, SaO_2_, and other routinely monitored simple parameters are of limited use in the detection of microperfusion and tissue oxygenation,^[26]^ NIRS monitoring provides useful additional information and may help in patient selection and in deciding between electrical cardioversion and pharmacological treatment.

## 4. Conclusions

In this case, significant instantaneous changes in NIRS were observed in the patient after sinus conversion, without obvious hemodynamic alterations of the systemic circulation and other monitoring values.

## Author contributions

**Conceptualization:** So Woon Ahn.

**Data curation:** Jin Ho Kim, So Woon Ahn.

**Writing – original draft:** He Won Hwang, Jin Ho Kim, So Woon Ahn.

**Writing – review & editing:** So Woon Ahn.
